# Adrenal angiomyolipoma: A rare entity

**DOI:** 10.4103/0970-1591.33734

**Published:** 2007

**Authors:** Rajesh Godara, M. G. Vashist, Sham L. Singla, Pradeep Garg, Jyotsena Sen, S. K. Mathur, Anshu Gupta

**Affiliations:** Department of Surgery, Post Graduate Institute of Medical Sciences, Rohtak, Haryana, India; * Department of Radiodiagnosis, Post Graduate Institute of Medical Sciences, Rohtak, Haryana, India; ** Department of Pathology, Post Graduate Institute of Medical Sciences, Rohtak, Haryana, India

**Keywords:** Adrenal, angiomyolipoma, extrarenal

## Abstract

Angiomyolipoma is apparently a part of a family of neoplasms that derive from perivascular epitheloid cells. It is a rare mesenchymal tumor, usually found in the kidney. Extrarenal angiomyolipoma is uncommon and the most common extrarenal site is the liver. Only two cases of adrenal angiomyolipoma are reported in English literature. Authors wish to add one more case to world literature. Because of its large size and symptomatic presentation of extremely rare tumor merits documentation.

## INTRODUCTION

Angiomyolipomas are rare mesenchymal tumors derive from perivascular epitheloid cells. These are commonly found in Kidney but extrarenal sites are also mentioned. Angiomyolipoma arising in adrenal is very rare entity, usually asymptomatic, diagnosed incidentally on radiological investigation of abdomen for other conditions. Angiomyolipoma are known diagnostic challenges to pathologists and a deligent search for adipocytes and abnormal blood vessels may help in confirming diagnosis.

## CASE REPORT

**Figure 1:**
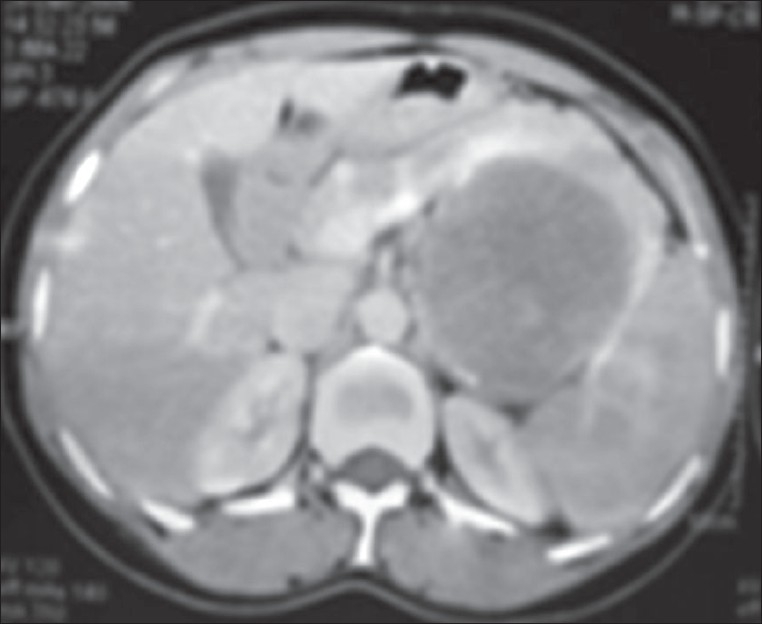
CECT abdomen showing well-defi ned non-homogeneous mass separate from left kidneyt

**Figure 2:**
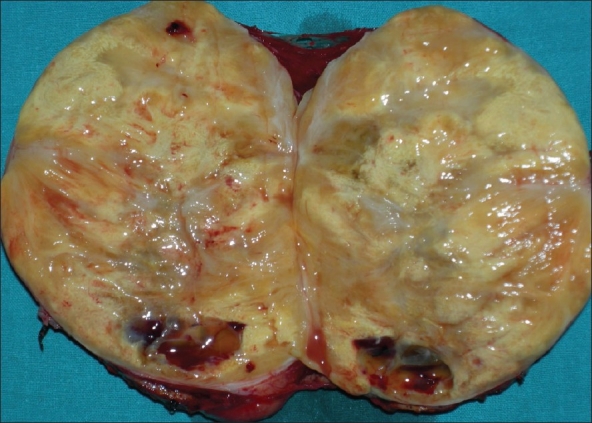
Cut section of removed mass

**Figure 3:**
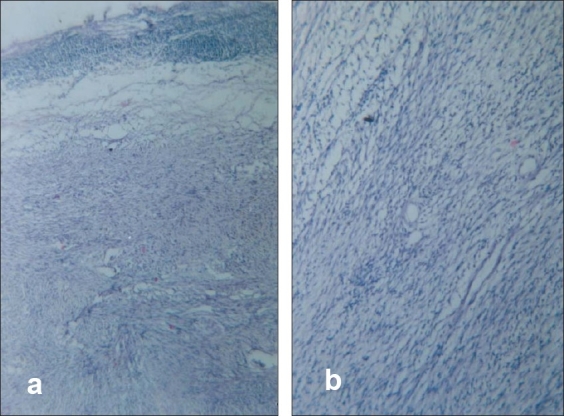
Photomicrograph (H and E staining) revealing features of angiomyolipoma with peripheral compressed adrenal cortical tissue

A 45-year-old female presented with epigastric discomfort off and on. Upper GI endosocopy was normal. Sonography for hepatobiliary system was normal but revealed a well-defined 15×12 cm mass in the retroperitoneum (incidentaloma). The CECT abdomen further defined the mass as of left adrenal origin and a possibility of adrenocortical tumor [Figure 1]. Laboratory investigations i.e., serum catecholamine, cortisol and urinary VMA were within normal limits. Exploratory laparotomy revealed a 15×12×10 cm mass, firm in consistency, quite separate from the left kidney with no definable left adrenal gland. On cut section mass was grey white and non-homogeneous in texture [Figure 2]. Histopathological examination revealed mature fat cells, smooth muscle fibers and thin-walled blood vessels with peripherally compressed adrenal cortical tissue suggestive of angiomyolipoma of adrenal [Figure 3]. Patient made an uneventful recovery and was normal at 18 months follow-up.

## DISCUSSION

An increasing number of incidental adrenal lesions have been reported recently, which may be attributable to the increasing use of better imaging techniques. Fatty tumors of the adrenal gland are uncommon and their features have received little attention in the literature. These include myelolipoma, lipomas, teratoma, liposarcoma and angiomyolipoma.[1] Angiomyolipoma of the adrenal gland is an extremely uncommon tumor detected incidentally at investigations for other reasons.

Angiomyolipomas are rare lesions, often arising in the kidney and are a part of a group of tumors with a diverse appearance known as PEComas (tumors of perivascular epitheloid cell origin). Angiomyolipoma most commonly occurs in the kidney. The next common site is the liver. Extrarenal angiomyolipomas are extremely rare and have been reported in the liver, colon, suprasellar region, small intestine, skin, intranodal, omentum, breast and adrenal gland.[1–5] Adrenal angiomyolipoma is extremely rare and only three cases have been reported, including the present case. One case was reported in the setting of tuberous sclerosis and other two were sporadic (one present case). The previous sporadic case was 8cm in size in a 46-year-old female. Both previously reported cases were in the left adrenal gland like the present one. The case in the setting of tuberous sclerosis was very small while the sporadic one was larger i.e., 8 cm. In our case the tumor size was (15×12 cm), the largest being reported. Angiomyolipomas predominately composed of smooth muscle cells are known diagnostic challenges to pathologists. They are often misdiagnosed as sarcomatoid carcinoma, carcinoma or sarcoma. Some of these tumors have malignant potential and recur locally. A diligent search for adipocytes and abnormal blood vessels may help in confirming the diagnosis.

Large angiomyolipomas even if asymptomatic should be removed to avoid complications like spontaneous rupture owing to the presence of abnormal elastin and poor vascularity in the tumor.[5] Nevertheless, follow-up is necessary because of atypical morphology.
